# Supplementary material for: Enzymatic Transglycosylation Features in Synthesis of 8-Aza-7-Deazapurine Fleximer Nucleosides by Recombinant E. coli PNP: Synthesis and Structure Determination of Minor Products
Source: Biomolecules. 2024 Jul 4;14(7):798. doi: 10.3390/biom14070798 (PMC11275124; doi:10.3390/biom14070798)
Supplement: Supplementary file 1 [file biomolecules-14-00798-s001.zip › biomolecules-3053216-supplementary.pdf]

Enzymatic Transglycosylation Features in Synthesis of 8-Aza-7-Deazapurine Fleximer Nucleosides by Recombinant *E. coli* PNP: Synthesis and Structure Determination of Minor Products

Barbara Z. Eletskaya [1], Anton F. Mironov [1,2], Ilya V. Fateev [1], Maria Ya. Berzina [1], Konstantin V. Antonov [1,*], Olga S. Smirnova [1], Alexandra B. Zatsepina [1], Alexandra O. Arnautova [1], Yulia A. Abramchik [1], Alexander S. Paramonov [1], Alexey L. Kayushin [1], Anastasia L. Khandazhinskaya [3], Elena S. Matyugina [3], Sergey N. Kochetkov [3], Anatoly I. Miroshnikov [1], Igor A. Mikhailopulo [4], Roman S. Esipov [1] and Irina D. Konstantinova [1,*]

[1] Shemyakin-Ovchinnikov Institute of Bioorganic Chemistry, Russian Academy of Sciences, Moscow 117997, Russia; fraubarusya@gmail.com (B.Z.E.); anton-mironov1999@inbox.ru (A.F.M.); ifateev@gmail.com (I.V.F.); berzina_maria@mail.ru (M.Y.B.); gescheites@gmail.com (O.S.S.); alex.zatsepina@gmail.com (A.B.Z.); 8818818@mail.ru (A.O.A.); ugama@yandex.ru (Y.A.A.); a.s.paramonov@gmail.com (A.S.P.); kayushin.alexej@yandex.ru (A.L.K.); aiv@mail.ibch.ru (A.I.M.); esipov@ibch.ru (R.S.E.)

[2] Institute of Biochemical Technology and Nanotechnology, Peoples' Friendship University of Russia Named after Patrice Lumumba, Miklukho-Maklaya St. 6, Moscow 117198, Russia

[3] Engelhardt Institute of Molecular Biology, Russian Academy of Sciences, 32 Vavilov St., Moscow 119991, Russia; khandazhinskaya@bk.ru (A.L.K.); matyugina@gmail.com (E.S.M.); snk1952@gmail.com (S.N.K.)

[4] Institute of Bioorganic Chemistry, National Academy of Sciences, Acad. Kuprevicha 5/2, 220141 Minsk, Belarus; imikhailopulo@gmail.com

* Correspondence: antonov.kant@yandex.ru (K.V.A.); kid1968@yandex.ru (D.K.)

Content	Page number
Scheme S1. Enzymatic transglycosylation of fleximer base 12 with the formation of reaction by-products **16**, **17**, **19**	SI-1
Figure S1. HPLC data of enzymatic reactions of synthesis nucleoside **15**, **16**, and **17**	SI-2
Figure S2. HPLC data of enzymatic reactions of synthesis nucleoside **18** and **19**	SI-3
Figure S3. HRMS spectrum of enzymatic ribosylation reaction	SI-4
Figure S4. HRMS spectrum of enzymatic deoxyribosylation reaction	SI-5
Figure S5-8. NMR spectra of compounds **16**	SI-6-8
Figure S9. Comparison of the H[^{13}C] HMBC NMR spectra of compounds **15** (A) and **16** (B)	SI-9-10
Figure S10-13. NMR spectra of compounds **17**	SI-11-13
Figure S14-18. NMR spectra of compounds **19**	SI-14-16
Quantum Chemical Analysis	SI-17
Figure S19. Ribosylation of heterocyclic base **12** at the pH 5-9 values of the reaction medium diverse pH values	SI-17
Table S1. Quantum Chemical Analysis of Nucleosides **16a** and **16b** *vs* **15** by PME and an *ab initio* amber FF 6-31G** methods	SI-18

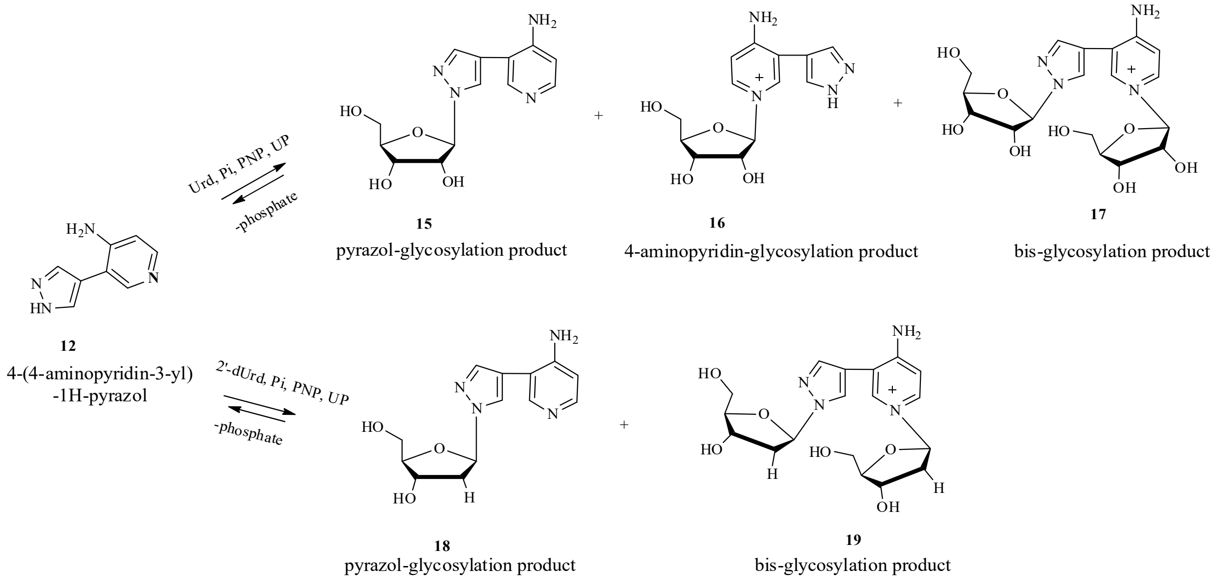

Scheme S1. Enzymatic glycosylation of fleximer base **12** with the formation of reaction by-products **16**, **17**, **19**.

HPLC data of enzymatic reactions.

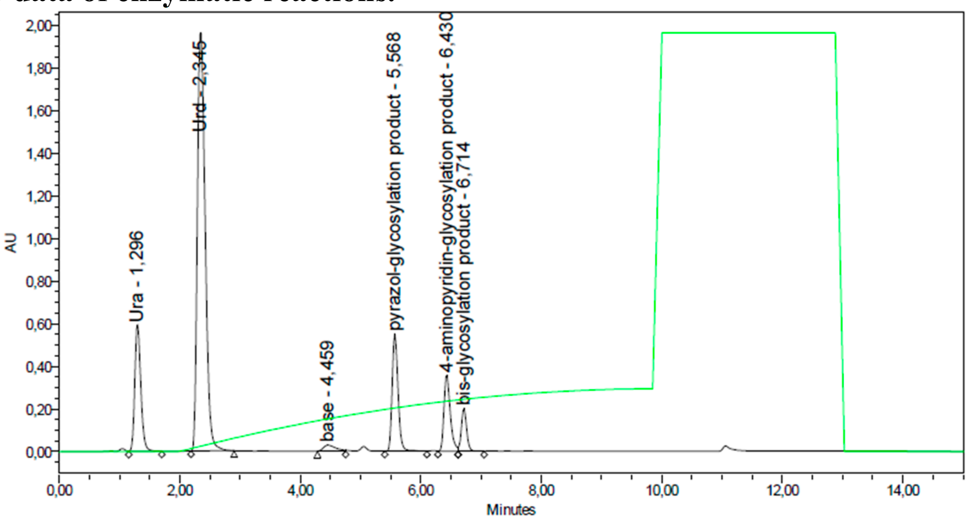

	Peak Name	RT (min)	Area (µV*sec)	% Area	Height (µV)	% Height
1	Ura	1,296	4099592	13,79	594009	16,06
2	Urd	2,345	17650985	59,38	1969274	53,24
3	base12	4,459	414165	1,39	28845	0,78
4	15	5,568	3738798	12,58	548613	14,83
5	16	6,430	2589720	8,71	358303	9,69
6	17	6,714	1232544	4,15	199763	5,40

Figure S1. HPLC reaction mixture producing enzymatic ribosylation reaction products (454 hours).

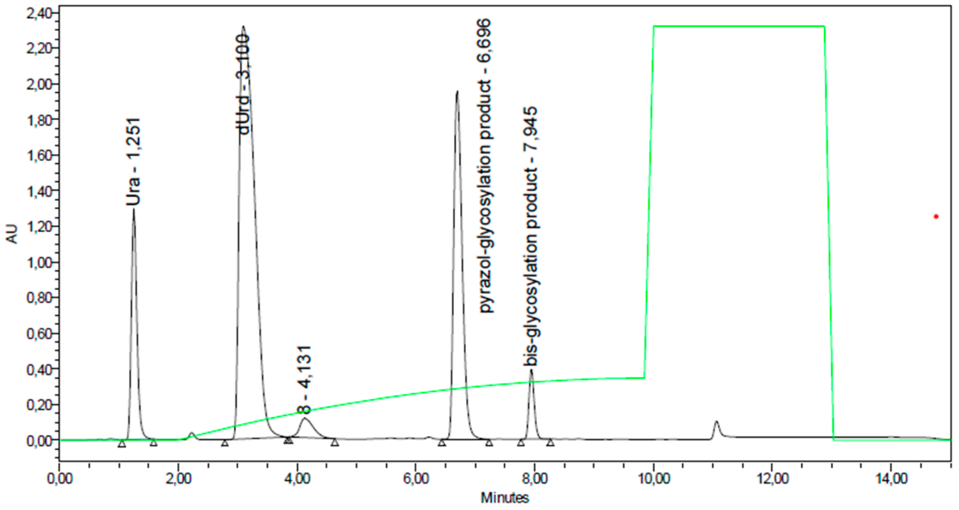

	Peak Name	RT (min)	Area (µV*sec)	% Area	Height (µV)	% Height
1	Ura	1,251	8395820	11,43	1289160	21,27
2	dUrd	3,100	41624246	56,65	2317827	38,24
3	12	4,131	1745301	2,38	107568	1,77
4	18	6,696	19309433	26,28	1956723	32,28
5	19	7,945	2399451	3,27	389526	6,43

Figure S2. HPLC reaction mixture producing enzymatic deoxyribosylation reaction products (3 hours)
Test reaction conditions: acceptor base (2 mM), dUrd or Urd (20 mM). 1.5 units of E. coli PNP were added to each reaction mixture. Reactions were carried out in 1.0 ml of 10 mM potassium phosphate buffer, pH 7.0, at 50°C.

Display Report

Analysis Info

Analysis Name	D:\Data\EM-85(LN98_Urd)_12_01_3404.d	Acquisition Date	7/30/2020 3:56:49 PM
Method	la_2.2_small.m	Operator	BDAL@DE
Sample Name	EM-85(LN98_Urd)	Instrument	compact 8255754.20088
Comment			

Acquisition Parameter

Source Type	ESI	Ion Polarity	Positive	Set Nebulizer	0.4 Bar
Focus	Active	Set Capillary	4500 V	Set Dry Heater	180 °C
Scan Begin	50 m/z	Set End Plate Offset	-500 V	Set Dry Gas	6.0 l/min
Scan End	3000 m/z	Set Charging Voltage	2000 V	Set Divert Valve	Source
		Set Corona	0 nA	Set APCI Heater	0 °C

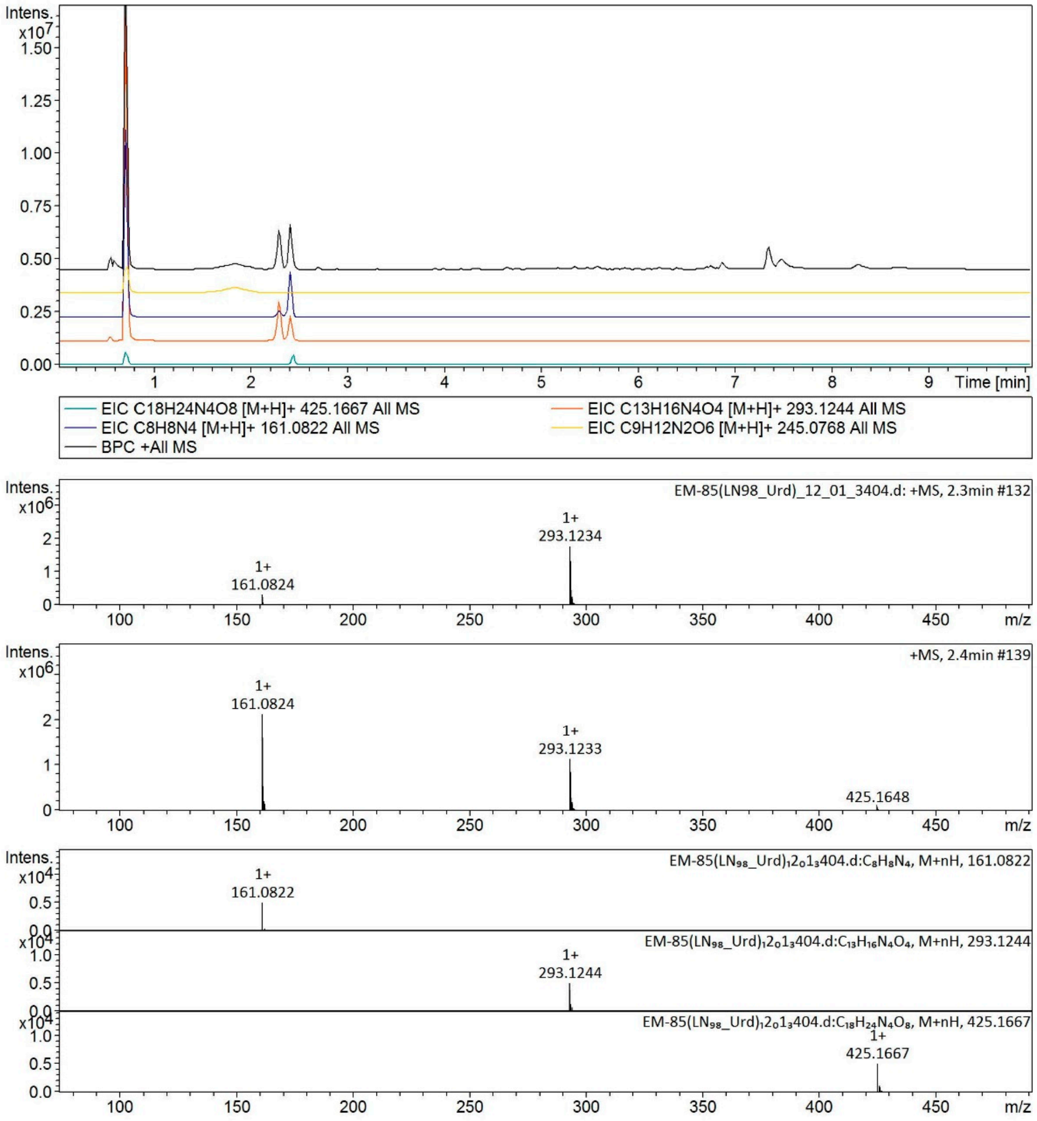

EM-85(LN98_Urd)_12_01_3404.d

Bruker Compass DataAnalysis 4.3 printed: 7/30/2020 5:02:01 PM by: BDAL@DE Page 1 of 1

Figure S3. HRMS spectrum of enzymatic ribosylation reaction.

Display Report

Analysis Info

Analysis Name	D:\Data\EM-84(LN98_2'dUrd)_11_01_3403.d	Acquisition Date	7/30/2020 3:45:10 PM
Method	Ia_2.2_small.m	Operator	BDAL@DE
Sample Name	EM-84(LN98_2'dUrd)	Instrument	compact 8255754.20088
Comment			

Acquisition Parameter

Source Type	ESI	Ion Polarity	Positive	Set Nebulizer	0.4 Bar
Focus	Active	Set Capillary	4500 V	Set Dry Heater	180 °C
Scan Begin	50 m/z	Set End Plate Offset	-500 V	Set Dry Gas	6.0 l/min
Scan End	3000 m/z	Set Charging Voltage	2000 V	Set Divert Valve	Source
		Set Corona	0 nA	Set APCI Heater	0 °C

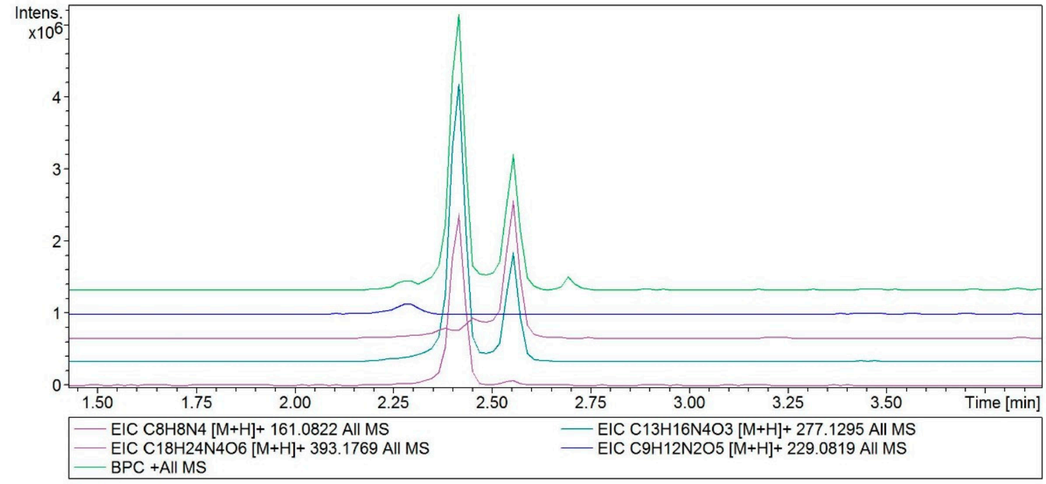

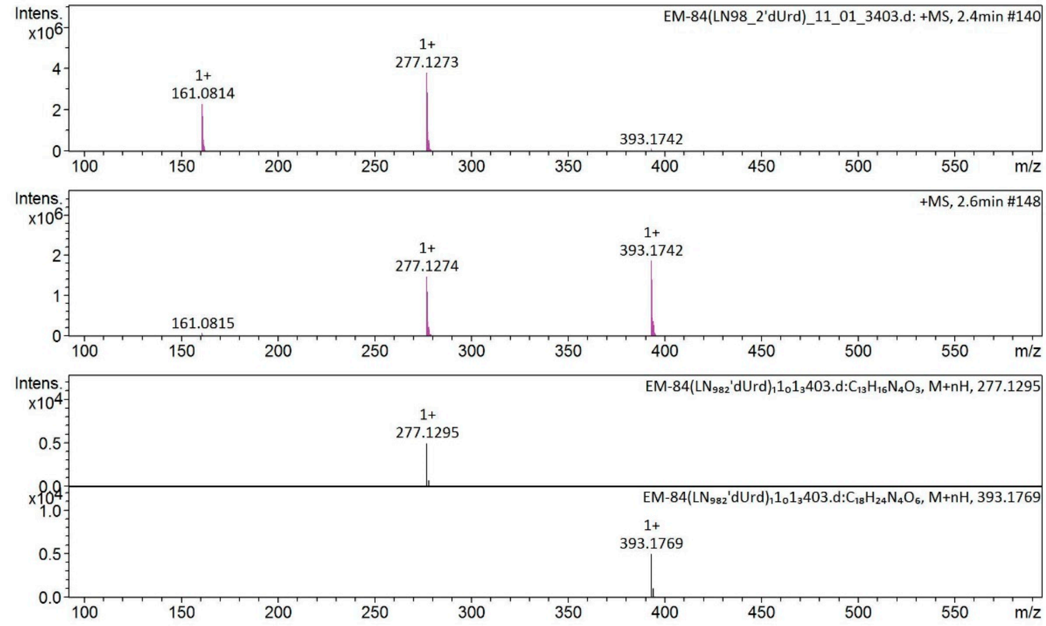

Figure S4. HRMS spectrum of enzymatic deoxyribosylation reaction.

NMR spectra of compounds 16

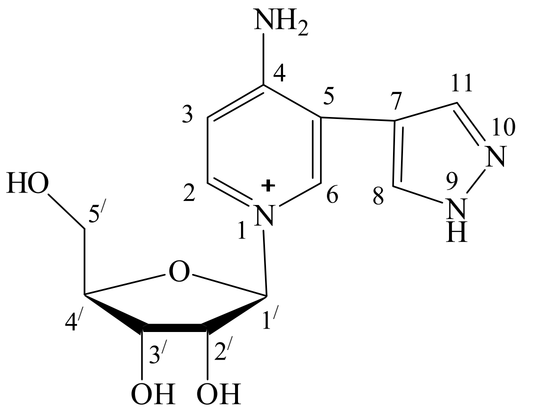

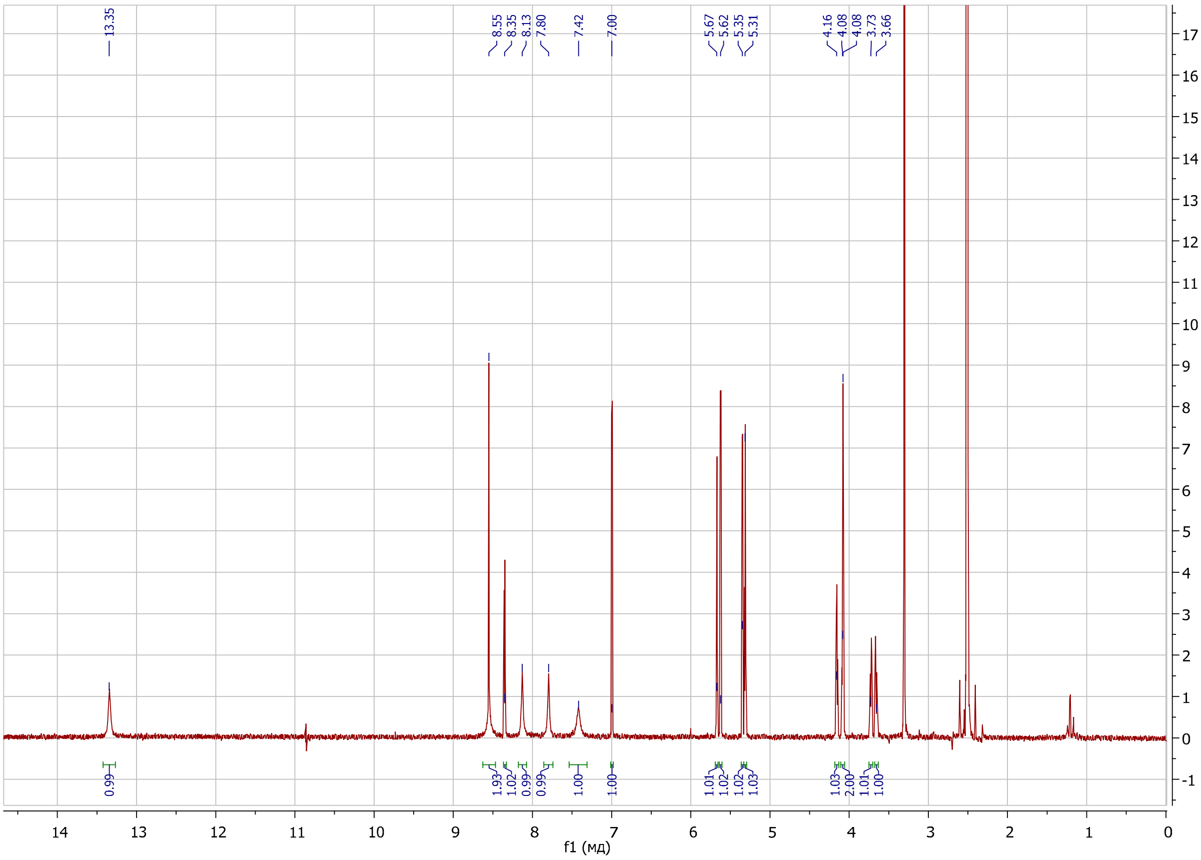

Figure S5. ^{1}H NMR spectrum of **16** in DMSO-d_6

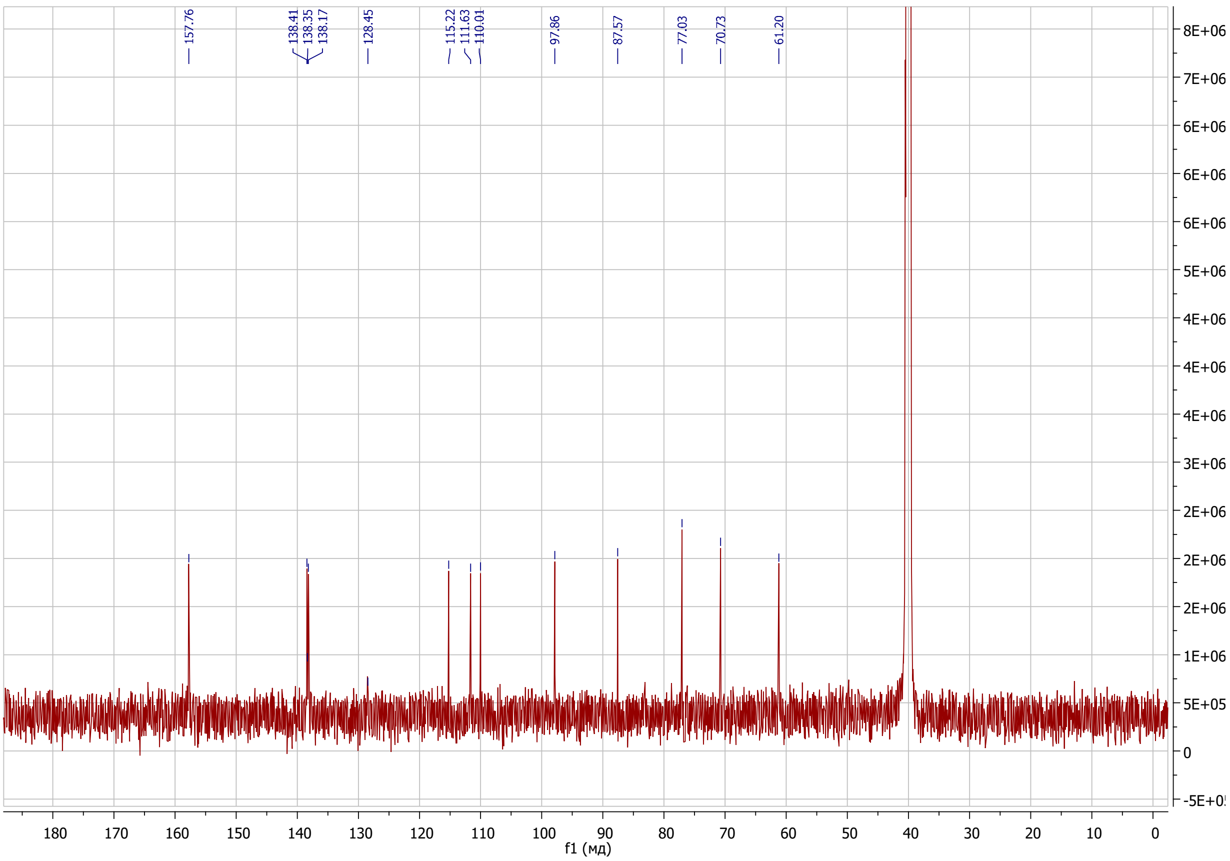

Figure S6. ^{13}C NMR spectrum of **16** in DMSO-*d₆*

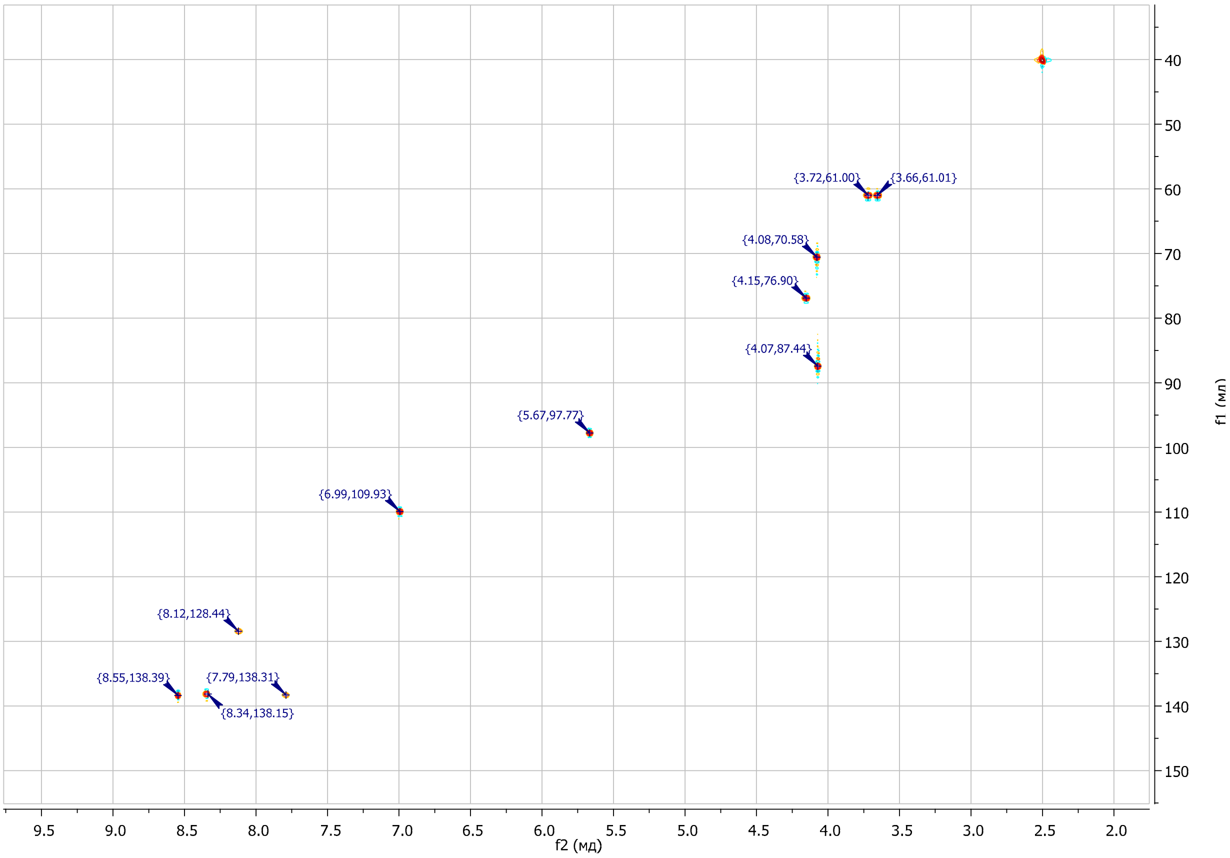

Figure S7. Superimposed ^{1}H-^{13}C HSQC spectra of **16** in DMSO-*d₆*

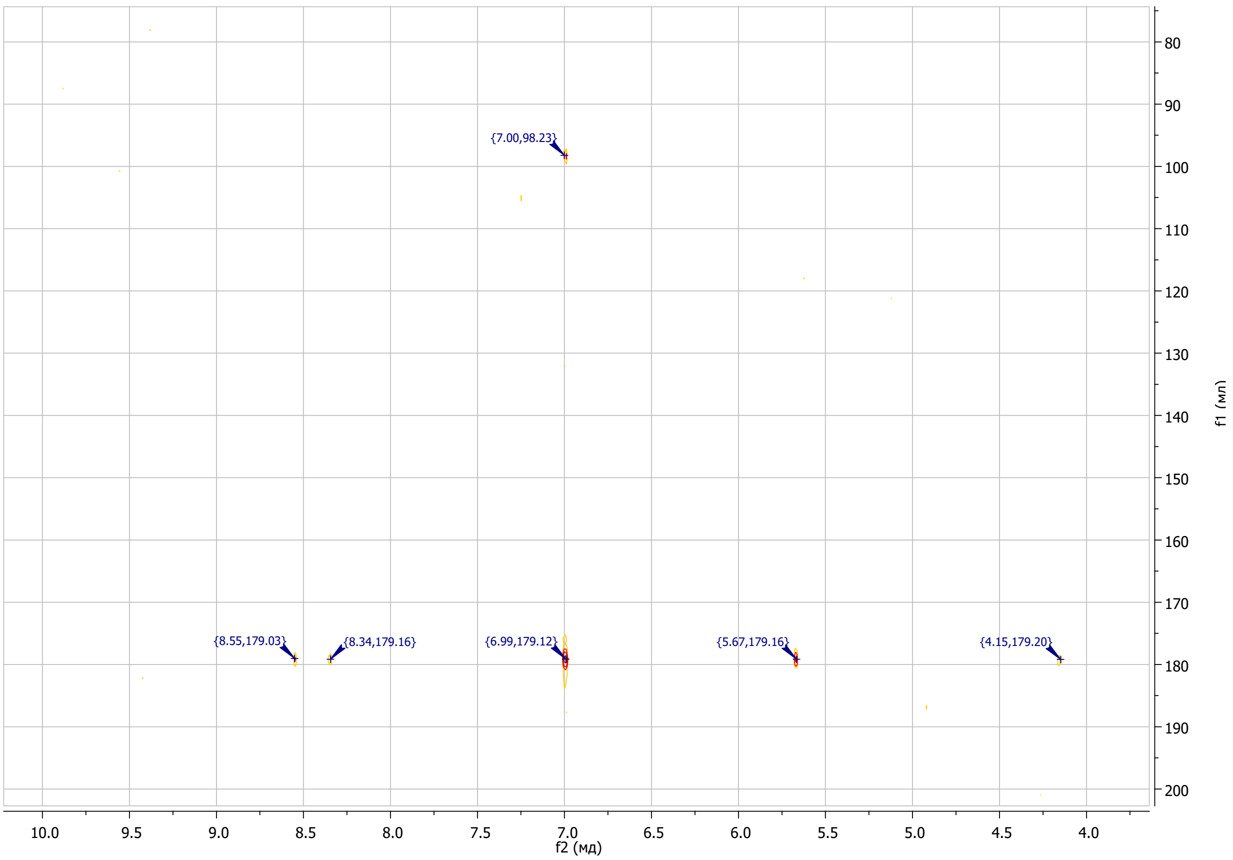

Figure S8. Superimposed ^{1}H-^{15}N HMBC spectra of **16** in DMSO-d_6

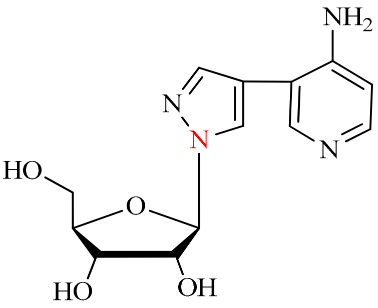

15

pyrazol-glycosylation product

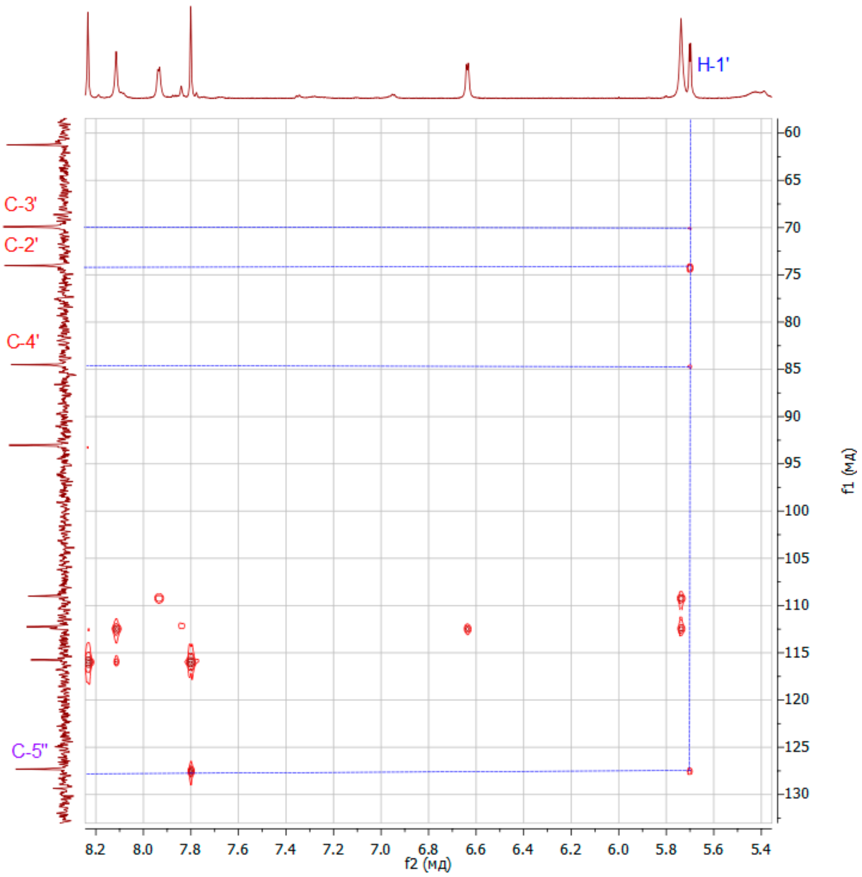

A

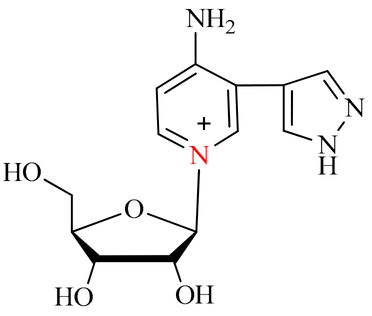

16

4-aminopyridin-glycosylation product

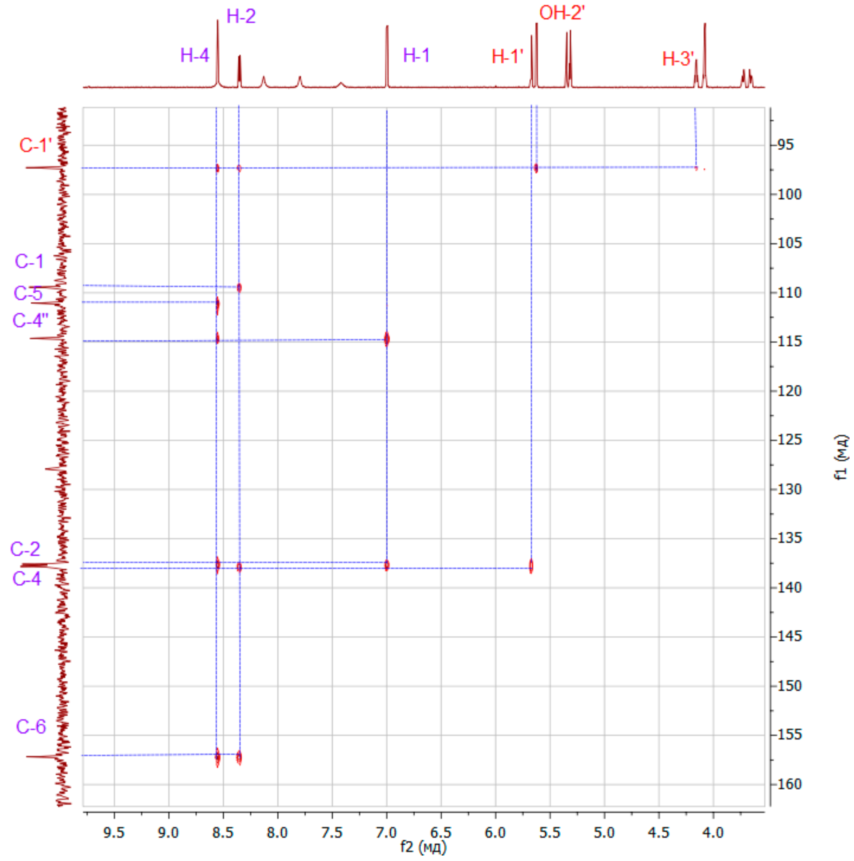

B

Figure S9. Comparison of H[^{13}C] HMBC NMR spectra of compounds **15 (A)** and **16 (B).**

NMR spectra of compounds 17

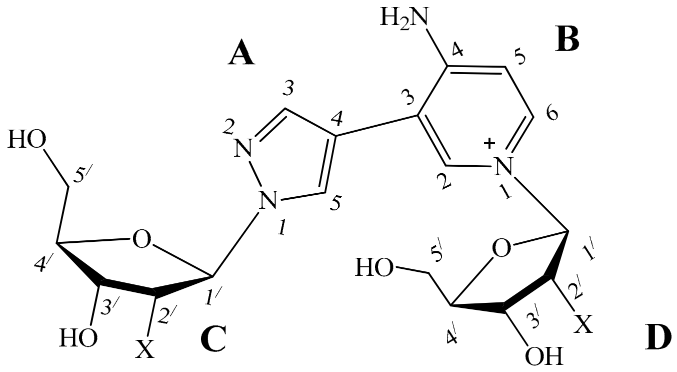

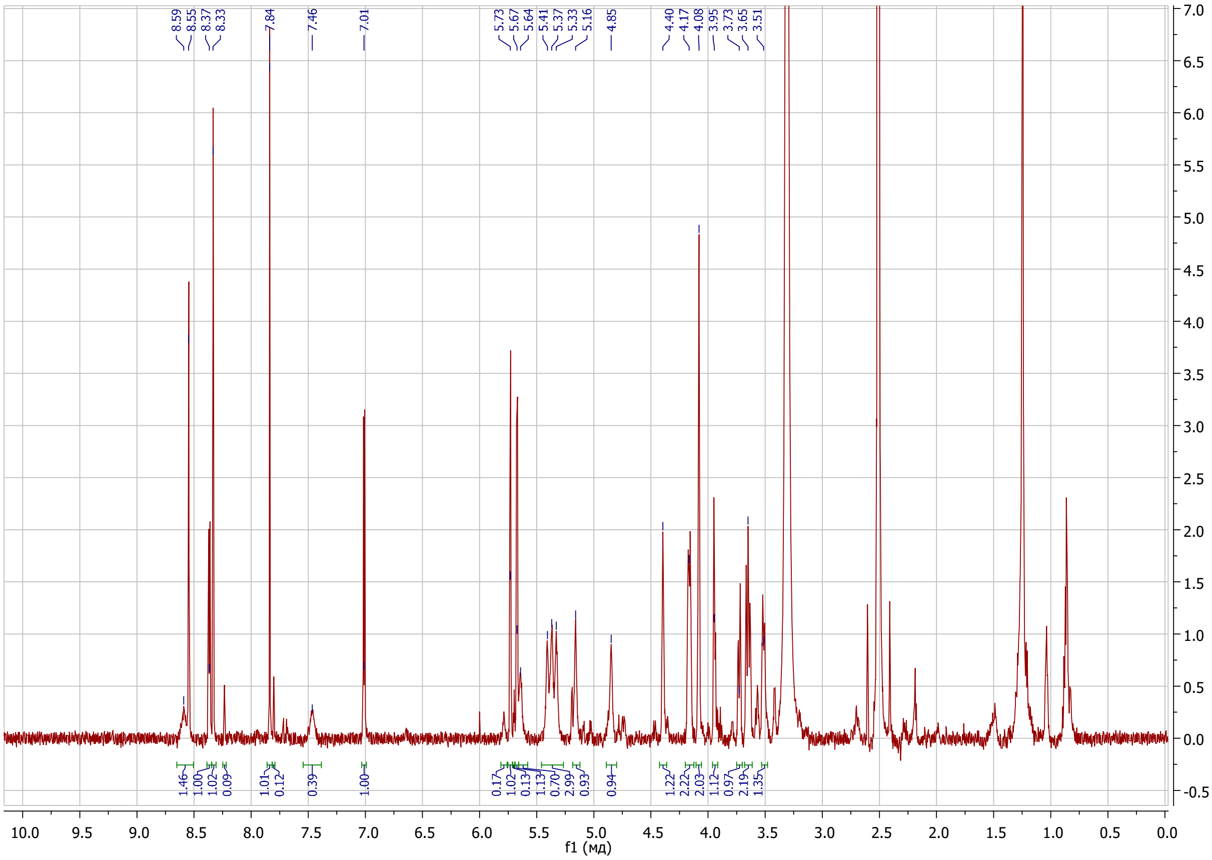

Figure S10. ^{1}H NMR spectrum of **17** in DMSO-d_6

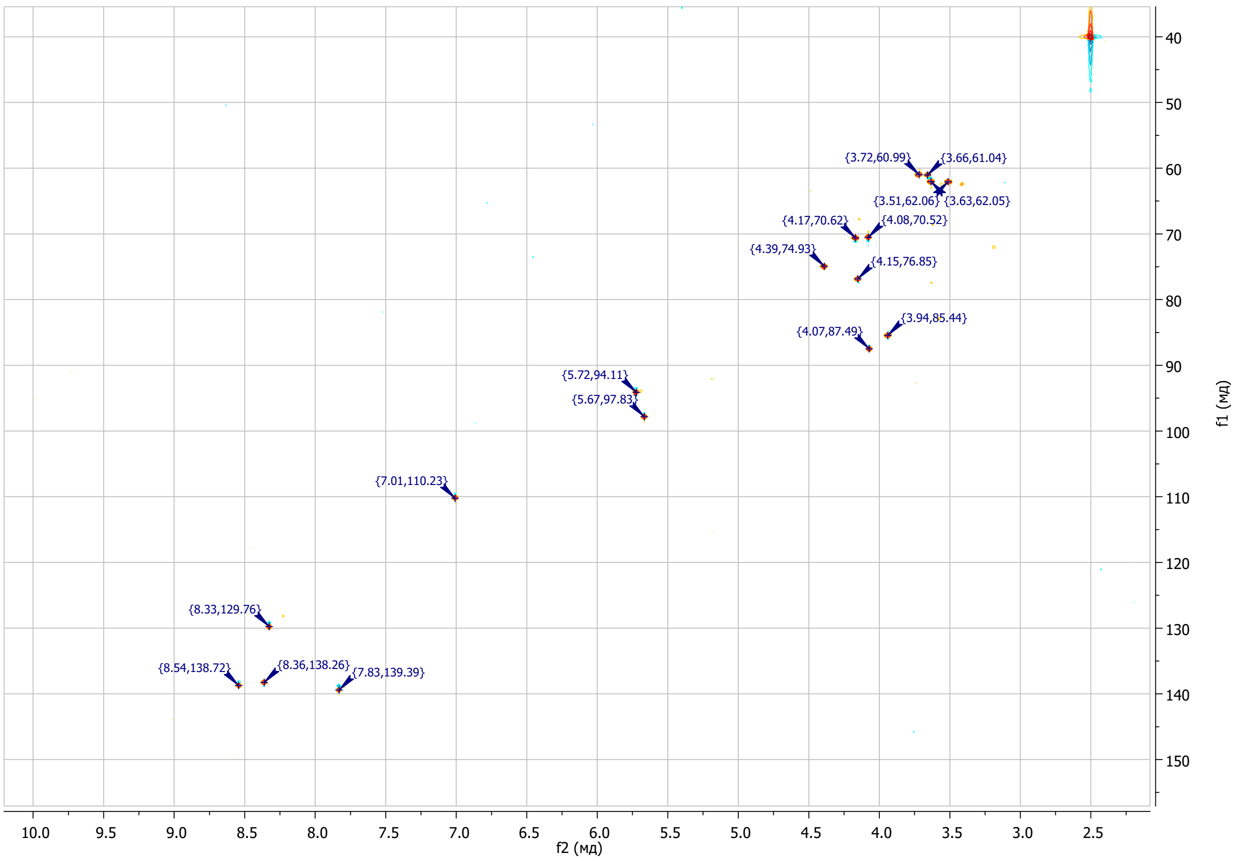

Figure S11. Superimposed ^{1}H- ^{13}C HSQC NMR spectrum of **17** in DMSO-*d*$_6$

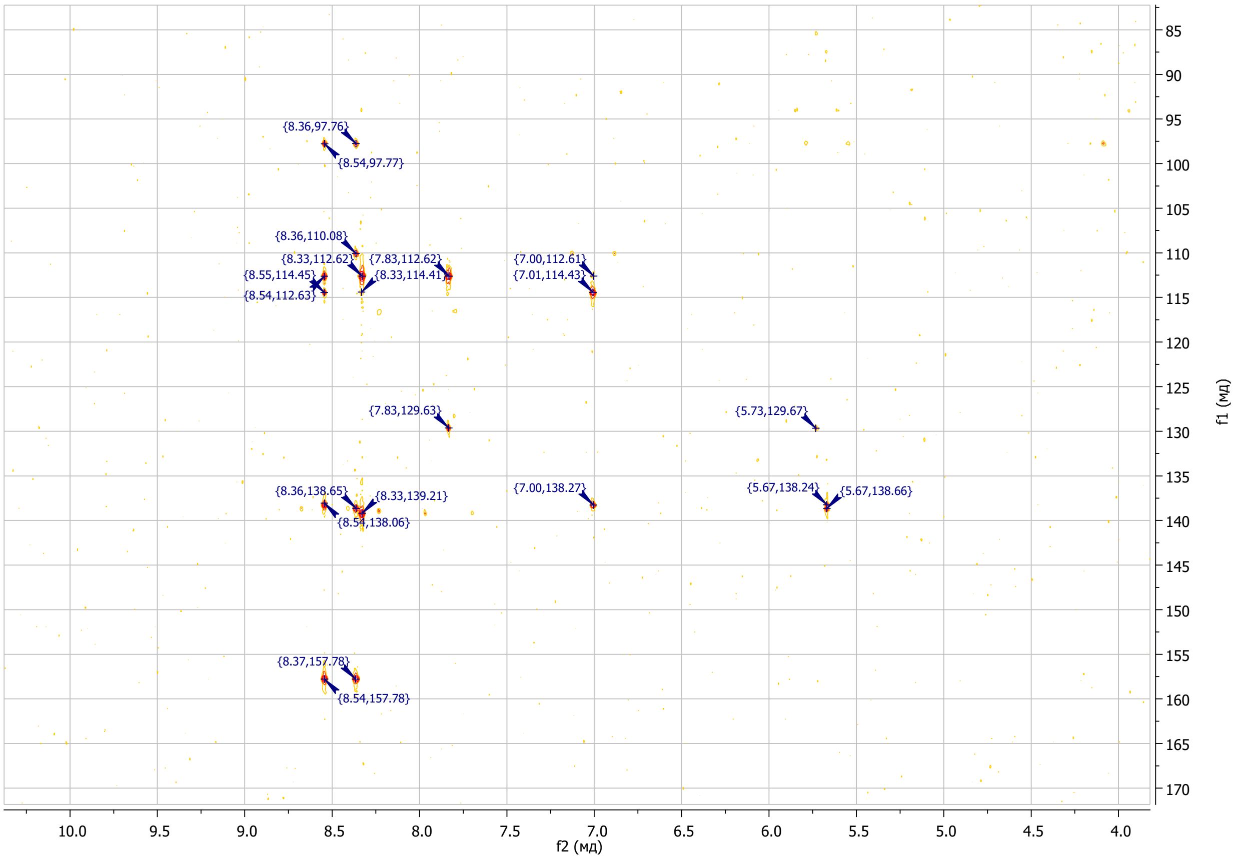

Figure S12. Superimposed ^{1}H- ^{13}C HMBC NMR spectrum of **17** in DMSO-*d*$_6$

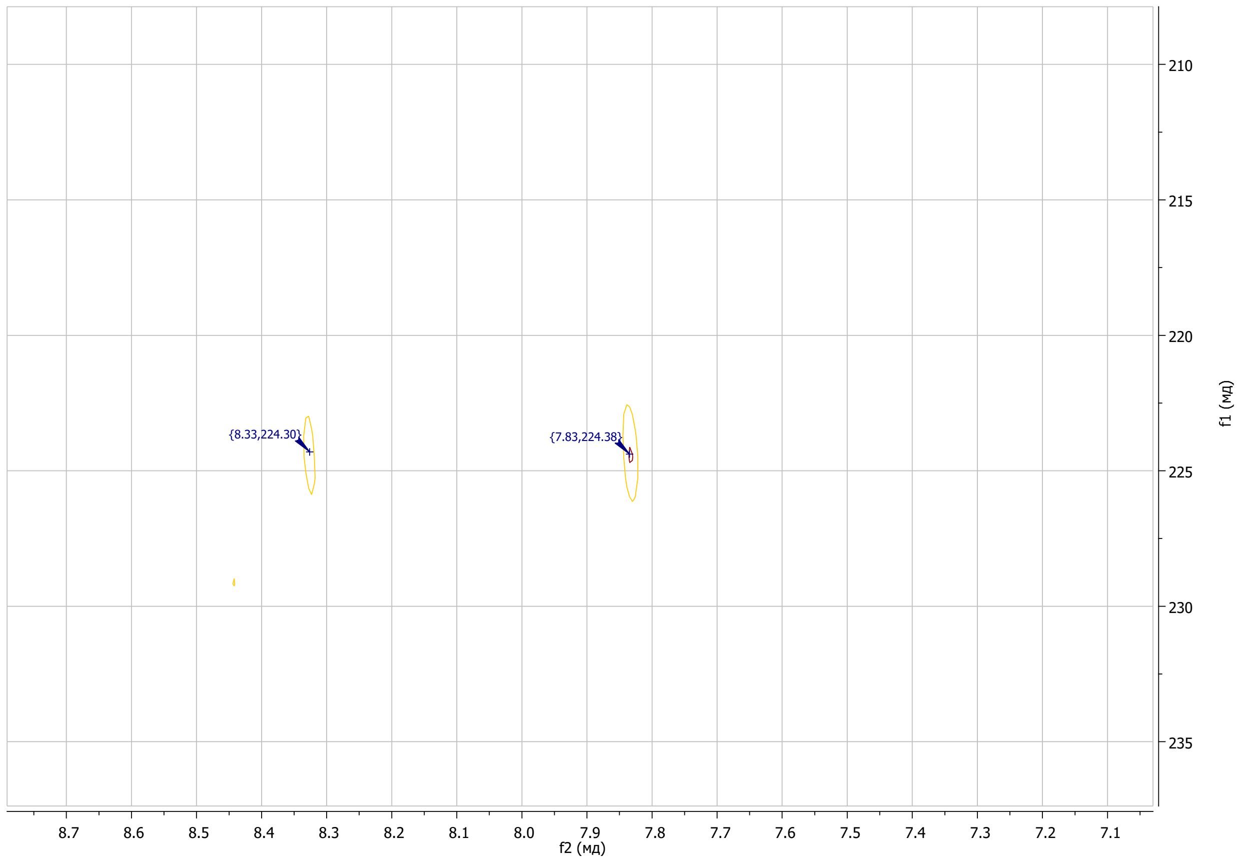

Figure S13. Superimposed ^{1}H-^{15}N HMBC NMR spectrum of **17** in DMSO-d_6

NMR spectra of compounds 19

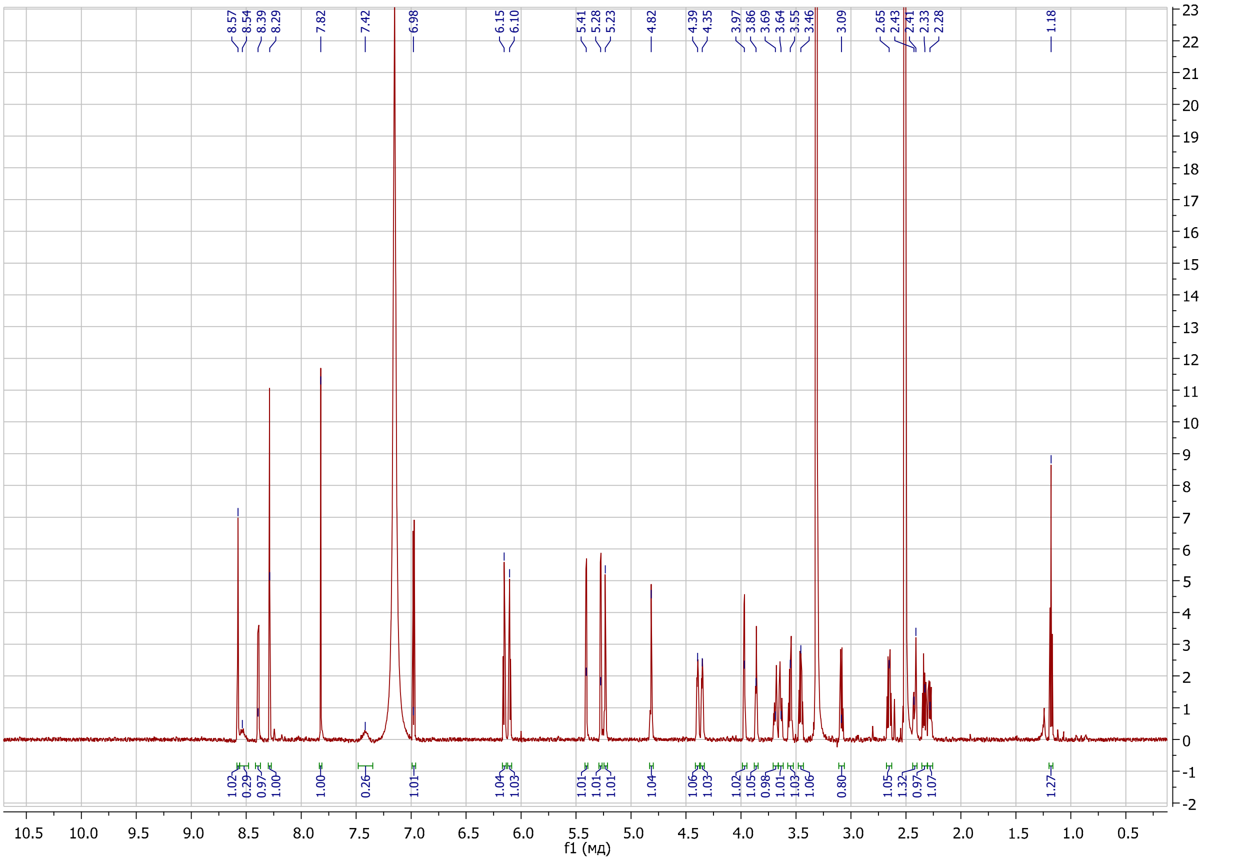

Figure S14. ^{1}H NMR spectrum of **19** in DMSO-*d$_6$*

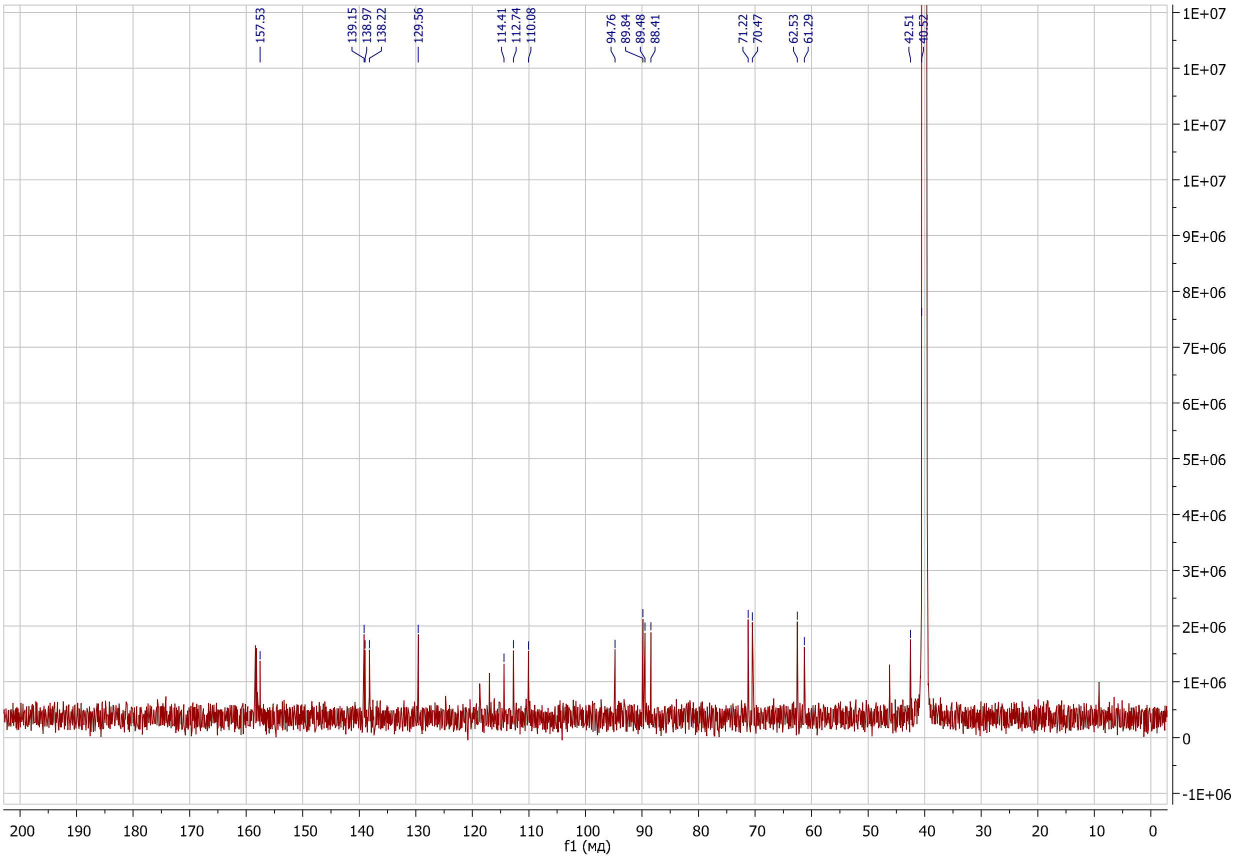

Figure S15. ^{13}C NMR spectrum of **19** in DMSO-*d$_6$*

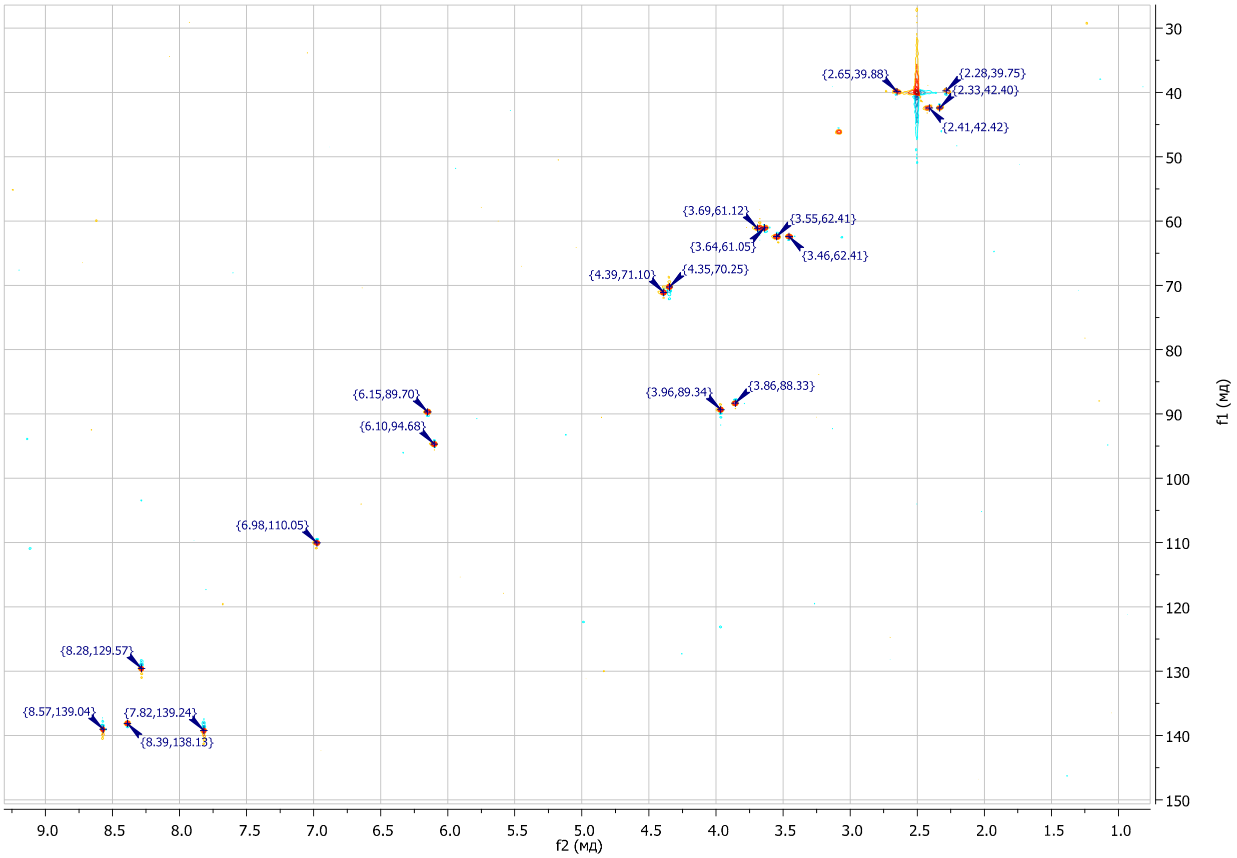

Figure S16. Superimposed ^{1}H-^{13}C HSQC spectra of **19** in DMSO-*d*$_6$

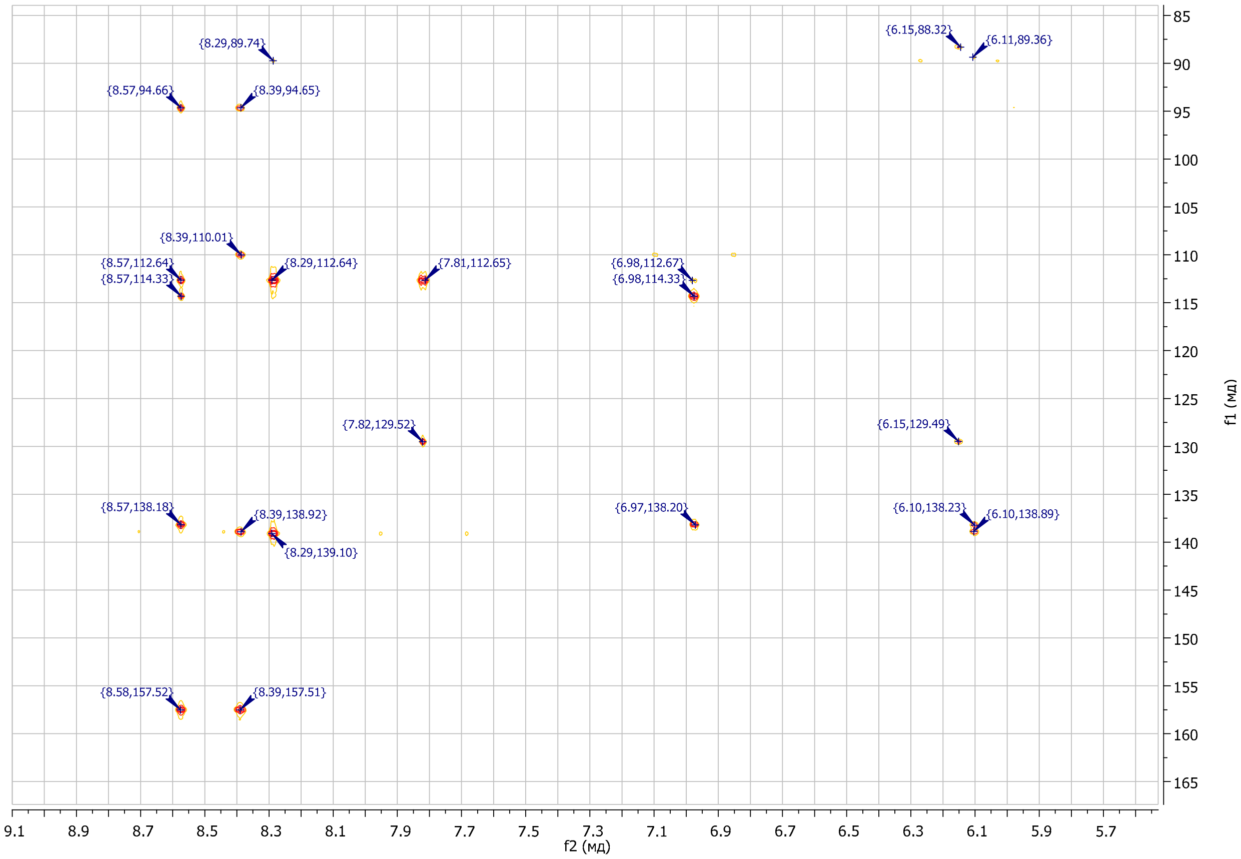

Figure S17. Superimposed ^{1}H-^{13}C HMBC spectra of **19** in DMSO-*d*$_6$

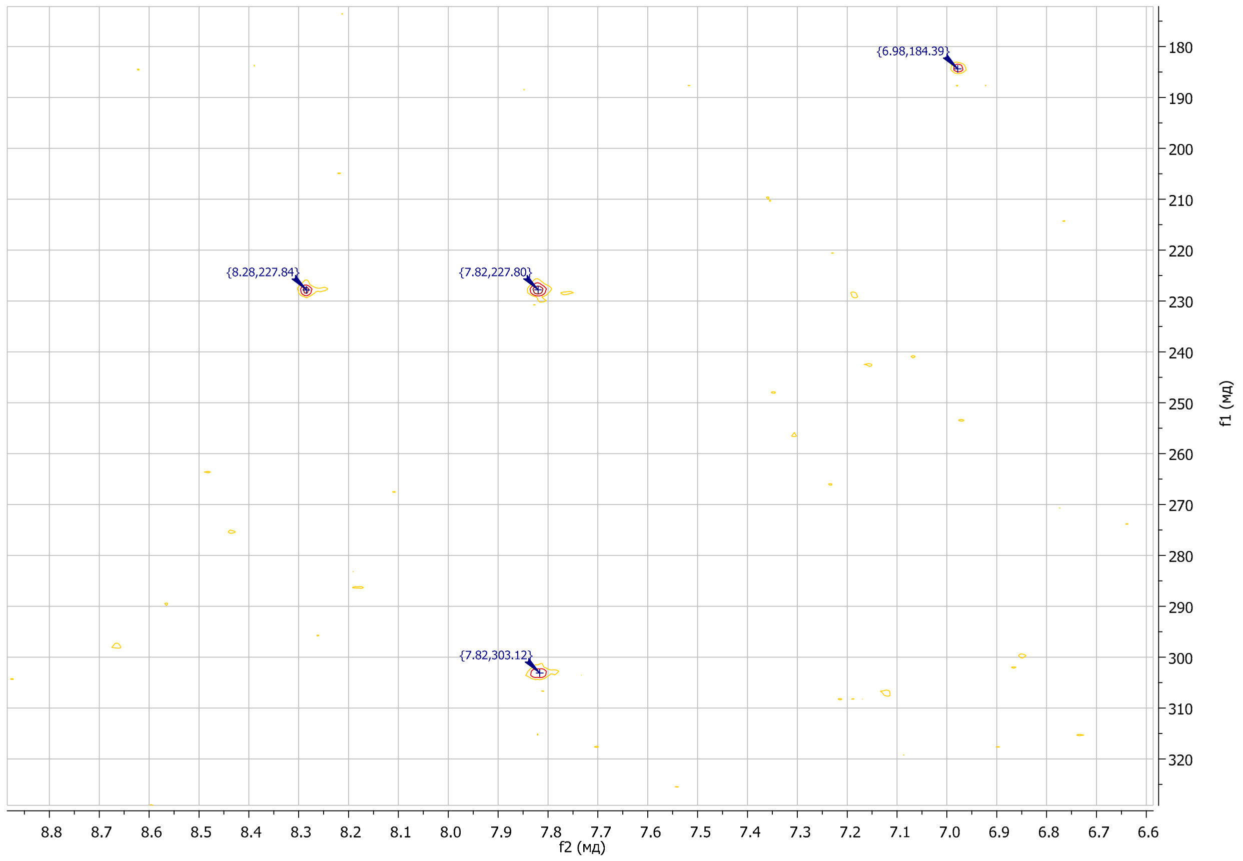

Figure S18. Superimposed ^{1}H-^{15}N HMBC spectra of **19** in DMSO-d_6

Quantum Chemical Analysis

Comparison of the partial charges of sp^2 nitrogen atoms of the pyrazole (N^1= -0.327 *e*) and pyridine (N^1 aver. -0.4140 *e*) fragments of base **12** convincingly testifies in favor of the superior nucleophilicity of the second, regardless of the method of substrate binding in the enzyme catalytic site. In spite of this, pyrazolic riboside **15** was predominantly formed, and in the case of 2-deoxyribosylation, the corresponding glycoside was not detected in the studied pH range.

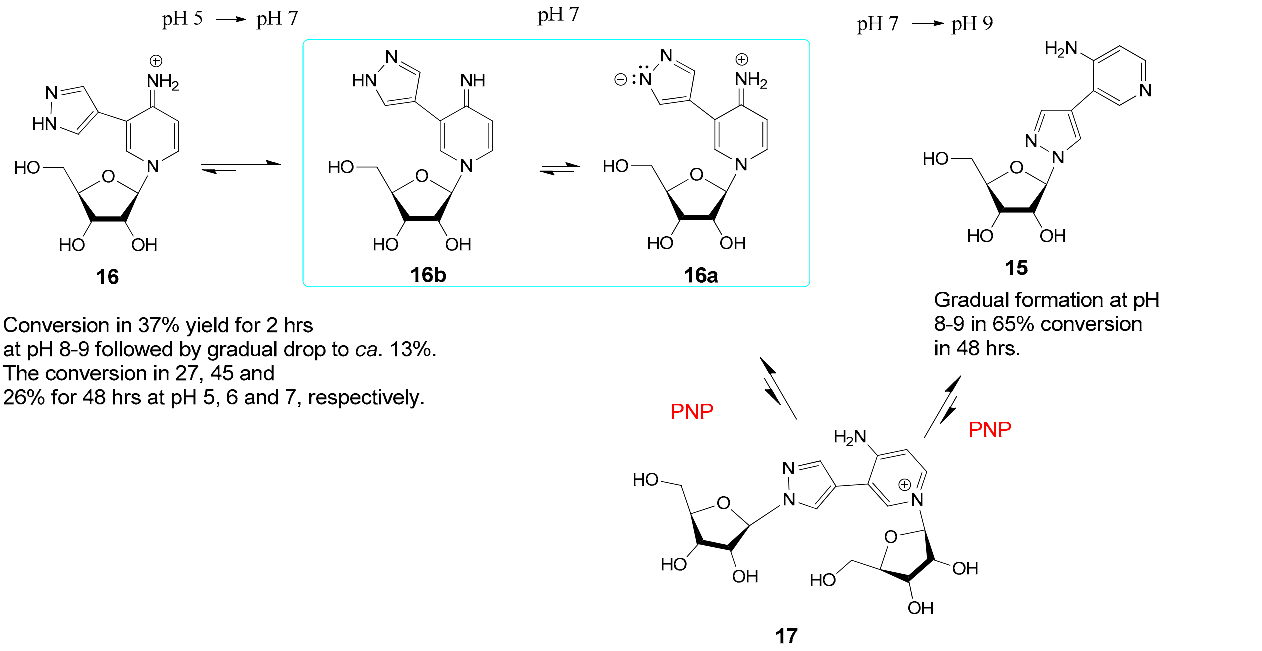

Figure S19. Equilibrium state of all the components involved in the base **12** ribosylation at the pH 5-9 values of the reaction medium.

Figure SI 19 shows the equilibrium state of all the components involved in the base **12** ribosylation process. Concerning the structure of riboside **16**. Based on the analysis of data from a number of studies on the structure of clitidine and related derivatives [53], we propose to consider three possible structures **16**, **16a** and **16b.** The reaction under study is of considerable interest from the point of view of the structure of the resulting nucleosides, primarily pyridine glycosides. With this aim in view, a quantum-chemical analysis of two possible structures, zwitterionic (**16a**) and so-called "free" (**16b**), was carried out in comparison with pyrazole glycosides, e.g. **15**. The probability of the existence of structures **16a** and **16b** arose as a result of the analysis of the chemical structures of the nucleoside antibiotic Clitidine and related compounds (Scheme 2). Data ore given in Table 2.

Table S1. Quantum Chemical Analysis of Nucleosides **16a** and **16b** *vs* **15** by PME and an *ab initio* amber FF 6-31G** methods.

**15** (PM3; RMS ‹0.1)<br>E_T = -83236.6 kcal/mol; 3′-exo; DM 3.983 Debye's; C1′-$N^1$1.4852 Å;	**16b** (PM3; RMS ‹0.1; Neutral molecule)<br>E_T = -83216.6 kcal/mol; 4`-endo; DM 6.066 Debye's; C1′-*N* 1.4916 Å;	**16a** (PM3; RMS ‹0.1; Zvitterionic struct.)<br>E_T = -83202.5 kcal/mol; 3`-exo; DM 14.69 Debye's; C1′-*N* 1.5058 Å;
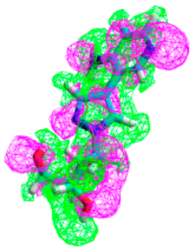	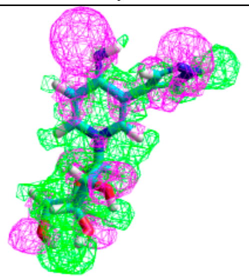	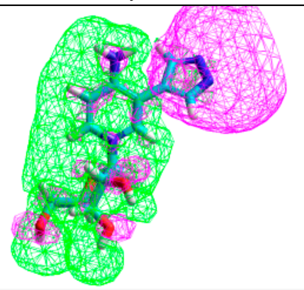
**15** (6-31G**; RMS ‹0.5)<br>E_T = -639 329.4 kcal/mol;<br>(ΔE_T -639 329.4 –(-639 302.8) = -26.6 kcal/mol);<br>C3`-exo; DM 4.305 Debye's<br>C1′-$N^1$1.44265 Å; (ΔÅ 1.4427 – 1.4383 = +0.0044);<br>H-Bond C5′OH..N^2 3.092(2.200) Å.<br>Δ*E* = -26.6 & 49.4 kcal/mol.	**16b** (6-31G**; RMS ‹0.5; Neutral molecule)<br>E_T = -639 302.8 kcal/mol;<br>(ΔE_T -639 302.8 –(-639 280.0) = -22.8 kcal/mol);<br>C3'-exo; DM 8.833 Debye's;<br>C1'-$N^1$1.4383 Å (ΔÅ 1.4656 – 1.4383 = -0.0273);	**16a** (6-31G**; RMS ‹1.0; Zvitterionic struct.)<br>E_T = -639 280.0 kcal/mol;<br>(ΔE_T -639 302.8 –(-639 280.0) = +22.8 kcal/mol);<br>C3'-exo; DM 19.95 Debye's;<br>C1'-N^1 1.4656 Å (ΔÅ 1.4656 – 1.4383 = +0.0273);
Coplanar orientation of two fragments!		

As follows from the data in **Table S1**, both methods of analysis used give close results, while the existence of a zwitterionic structure seems unlikely in the case of compound **16a**. Thermodynamically, the most probable molecule is a pyrazole nucleoside, which follows from the analysis data and agrees with the experimental results.
